# Learning from health information challenges in the Central African Republic: where documenting health and humanitarian needs requires fresh approaches

**DOI:** 10.1186/s13031-021-00405-1

**Published:** 2021-09-16

**Authors:** Anna Kuehne, Leslie Roberts

**Affiliations:** 1 Médecins Sans Frontières, Berlin, Germany; 2 Médecins Sans Frontières, London, UK; 3 Columbia University, New York, NY USA

## Abstract

The Central African Republic (CAR) is one of the world’s poorest and most fragile countries. Maybe there is no nation on the planet where the official health statistics are so poor. Evidence presented in this Conflict and Health themed collection to document humanitarian needs in CAR, suggests that UN statistics dramatically under-estimate the birth and death rates in conflict settings. To be current and valid, health indicator data in violent settings require more frequent measurement, more triangulation and granular exploration, and creative approaches based on few assumptions. In a world increasingly dependent on model driven data—data often inaccurate in conflict settings—we hope that this collection will allow those service providers and researchers operating in CAR to share their work and help us better learn how to learn. We particularly invite research from professionals working in CAR that documents humanitarian needs and presents indicators of population health where official estimates might not articulate the true extent of the health crisis.

## Background

The world over, estimates of mortality and birth rates as well as health service access and coverage data are used as indicators for progress towards national public health targets and the UN Sustainable Development Goals (SDGs) [1]. However, the quality of these data is far from uniform across all countries. While trustworthy data from civil registration, health services, recent representative countrywide surveys or well-modelled data is available for most high-income countries, data sources become scarce and estimates are associated with substantial uncertainty in many low-income countries. The required indicators are often least reliable in conflict settings where health information may be inaccurate, incomplete, or manipulated [2]. This is concerning as health information data is used to allocate funding and to monitor humanitarian needs by governments, UN agencies and donors. This is particularly problematic as trends suggest conflict affected countries are becoming a larger fraction of the world populations not achieving the SDGs [3].

### Obstacles to reliable health information in a conflict setting: the example of CAR

The Central African Republic (CAR) is one such setting where multiple obstacles to documenting humanitarian needs exist. CAR experienced a deadly civil war in 2013–2014, and has since been affected by frequent upsurges of violence. Approximately 70% of the countryside and one third of the population have been outside of the government’s control for more than a decade [4]. Despite repeated peace deals between the 14 non-state armed groups operating in CAR, armed groups continue to threaten the life and safety of the population throughout the country [4]. CAR is perhaps the world’s poorest and most fragile country [5, 6]. At the same time, year after year, CAR is among the countries furthest from reaching their UN appeal targets [7].

CAR is experiencing most of the difficulties to good data that arise in many conflicts, but is unique in that it has so many health information barriers, and that these barriers have in many ways remained in place for decades. Because so much of the country is inaccessible to the Ministry of Health, dozens of different non-governmental organizations (NGOs) with different funding streams and protocols provide health services, and document those services and health conditions differently. Still, a substantial proportion of the population has no access to health care at all. Additionally, contributing to the scarcity of data is the de facto absence of civil registration data and the lack of countrywide representative surveys since the start of the civil war in 2013. Since then, the country experienced repeated waves of violence, resulting in thousands of people being displaced—sometimes repeatedly throughout the years—and an unknown number of dead civilians [4, 8]. Most importantly for global health policy; there may be no nation on the planet where the official health statistics are so poor.

The UN estimates a crude mortality rate (CMR) of 11.3/1,000/year, and a birth rate of 34.5 births per 1,000 per year population in 2020 [9]. The UN mortality statistics indicated a steady decline of crude and under five mortality rates (CMR and U5MR) over decades [9], even through the deadly civil war period in 2013–14 when more than one fifth of the population fled their homes and bodies covered the streets of the capital city [10]. This reported steady drop in all cause CMR and U5MR from 2012 to 2013 to 2014, provides a striking insight into the inability of modelling-based data to capture the reality in this conflict-affected setting [9].

Even more stunning is the UN statistics on birth rates for CAR that display a 35-year unfaltering decline with the post-2015 birth rate of approximately 3.5% per year [9]. This birth rate is implausible given that the two studies presented in this series, previous estimates by NGOs and a Multiple Indicator Cluster Survey (MICS) in 2019, have all found 20–24% of the living population is less than 5 years old [11–13]. If 3.5% of the population were newborns each year and none of them ever died or left the country, one would expect slightly over five times that fraction to be less than 5 years of age, i.e. 17.5% of the population. But, for 22% of the population to be less than five in a setting with high child mortality, one would need an annual birth rate well over 5%. Compared to mortality, the birth rate is exceptionally easy to record. It does not vary as much from month to month. People have less incentive to lie or hide births. Moreover, birth rates are used for many health system planning and reporting purposes. This underestimate by at least one third of all births implies that all indicators based on this denominator—such as vaccination coverage, and attended birth rate, and a host of other process indicators on which the SDGs are based—look far better than they really are.

This under-reporting of the birth rate and the death rate in CAR explains the low rate of population growth, while at the same time, having the effect of masking approximately 100,000 deaths per year. There are only five million people living in the CAR. Wars in Yemen and Syria are more central to geopolitical struggles. The refugees from the crisis in CAR are not overwhelming the borders of any wealthy state. But CAR may be the pinnacle of how health service provision and health information collection are failing in our present day world.

### UN health statistics in CAR and beyond

UN health estimates are usually based on modelling using census data (the last of which was in 2003) and other surveys such as MICS or Demographic and Health Surveys (DHS). After the 2010 MICS, the nation went for nearly a decade without any update. Hence, this impacts modelling validity [14]. The problem of poor health statistics in conflict settings is relevant beyond CAR. Concern has arisen that so much of the global health data on which policy and spending is based is formed with models, often using inputs that are themselves model outputs [15]. Conflict settings often involve dramatically and rapidly changing health conditions over space and time. Areas inaccessible or out of government control complicate the establishment of meaningful health information. Additionally, models usually assume that things change in a predictable manner and that common trends can be identified for the overall national population – assumptions that do often not hold true in conflict settings. Using outdated and potentially non-representative data as model inputs and applying assumptions that might not reflect the reality of a complex conflict creates estimates that may be very misleading. This seems to be the case in CAR.

## Conflict and Health series about CAR: documenting humanitarian needs in silent crises

In two of the first articles in the Conflict and Health series to document humanitarian needs in CAR, Robinson et al. in Ouaka Prefecture and Wol et al. in Ouham Pende Prefecture have measured a CMR four times higher than the official national rate, and an U5MR much higher than previous estimates [16, 17]. These are two examples of surveys, among a long list that include two nationwide surveys sponsored by UNICEF, that found mortality rates two to six times higher than the official model-driven UN mortality estimates [11, 12, 18–23]. At the same time, the documented birth rates and proportions of children under 5 years were much higher than the official rate, consistent with findings of other relief agencies [11, 12].

These first two articles provide just two examples of approaches to generate health data in conflict settings. To be current and valid, health indicator data in violent settings require more frequent measurement, more triangulation and granular exploration, and creative approaches based on few assumptions. This series is an attempt to allow those service providers and researchers operating in CAR to share their work and help us better learn how to learn. We particularly invite research from professionals working in CAR that documents humanitarian needs and presents indicators of population health where official estimates might not articulate the true extent of the health crisis. We hope that having a series in Conflict and Health will contribute to a more nuanced understanding of how inconsistent with human survival the conditions are in CAR, and bring attention to the crisis in a way that past isolated studies have not. It is also hoped that it will generate discussion on challenges with official health estimates in the Central African Republic as well as other similarly precarious conflict-affected countries.

## Data Availability

Not applicable.
